# Supplementary material for: Synthesis of Bithiophene-Based D-A1-D-A2 Terpolymers with Different A2 Moieties for Polymer Solar Cells via Direct Arylation
Source: Polymers (Basel). 2019 Jan 2;11(1):55. doi: 10.3390/polym11010055 (PMC6402016; doi:10.3390/polym11010055)
Supplement: Supplementary file 1 [file polymers-11-00055-s001.pdf]

Synthesis of bithiophene-based D-A1-D-A2 terpolymers with different A2 moieties for polymer solar cells via direct arylation

Huang jinfeng, Lin Zhenkun, Feng Wenhuai, Wang Wen*

Fujian Key Laboratory of Polymer Materials, College of Chemistry and Materials Science, Fujian Normal University, Fuzhou 350007, China.

* Correspondence: wangwen@fjnu.edu.cn

1.1 Synthesis of monomer (4,7-bis-(5-bromo-4-hexylthiophen-2-yl)-benzo[1,2,5]selenadiazole)

4, 7-dibromo-2, 1, 3-benzoselenadiazole was purchased from SunaTech Inc. The other reagents and starting materials were purchased from commercial sources. In the free-water and free-oxygen conditions, the solvent must be corresponding treatment with distillation and deoxidization.

4,7-bis-(5-bromo-4-hexylthiophen-2-yl)-benzo[1,2,5]selenadiazole

Compound (4, 7-dibromo-2,1,3-benzoselenadiazole) (0.76 g, 2.24 mmol), 2-bromo-3-hexylthiophene (1.16 g, 4.68 mmol), catalyst $Pd(OAc)_2$ (5 mol%), tricyclohexylphosphonium tetrafluoroborate (10 mol%), K_2CO_3 (0.32 g, 2.34 mmol) and pivalic acid (0.18 g, 1.17 mmol) were transferred to single-neck bottle under an atmosphere of nitrogen. The blend solutions (DMAc (5 mL) /p-xylene (5 mL)) were added and the mixture was refluxed at 110 ^{0}C for 48 h. After cooling to room temperature, the solvent was removed, and 0.39 g yellow solid was obtained in 25.8% yield by silica gel column chromatography. ^{1}H NMR (400 MHz, $CDCl_3$): δ_{ppm} 7.76 (d, 2H), 7.68 (d, 2H), 2.62 (m, 4H), 1.18-1.62 (m, 16H), 0.90-0.88 (m, 6H). Elemental analysis: Calcd for $C_{26}H_{30}Br_2N_2S_2Se$ (%): C, 46.37, H, 4.49, N, 4.16. Found (%): C, 46.85, H, 4.28, N, 4.23. Mass (m/z) [M^+]: Calcd for $C_{26}H_{30}Br_2N_2S_2Se$ 673.43. Found 672.89.

1.2 GPC traces and ^{1}H NMR spectra for Polymers

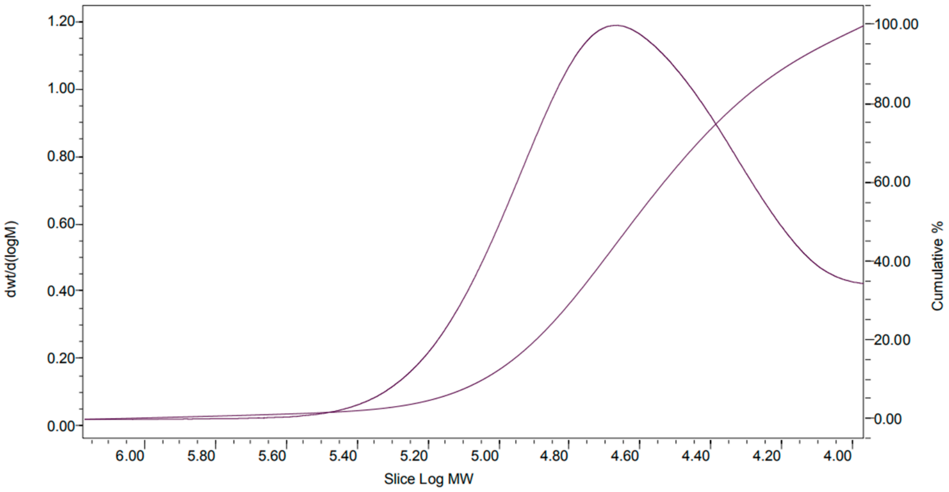

GPC Results

	Dist Name	Elution Volume (ml)	Retention Time (min)	Adjusted RT (min)	Mn	Mw	MP	Mz	Mz+1
1		17.190	17.190	17.190			2042602		
2		23.773	23.773	23.773	32368	64684	47797	237304	749667

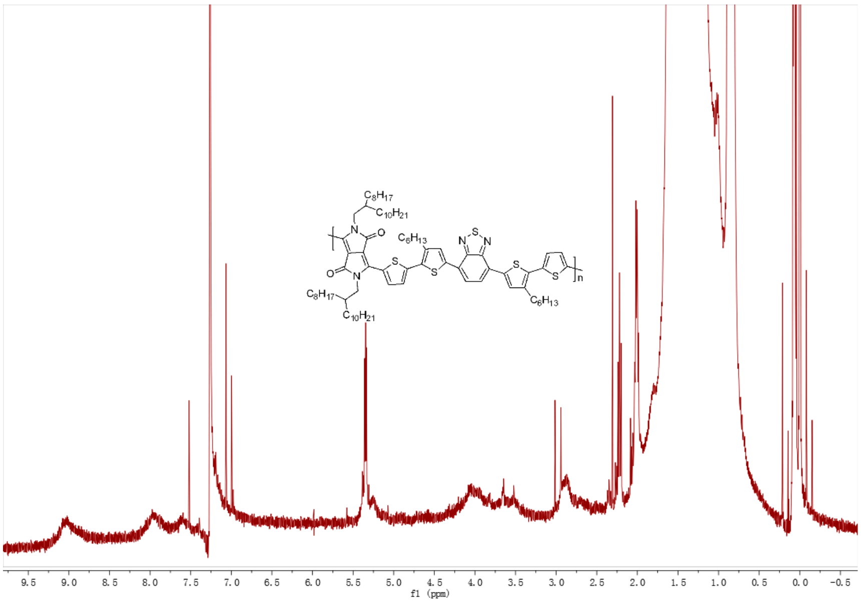

Figure S1 GPC traces and ^{1}HNMR of P1

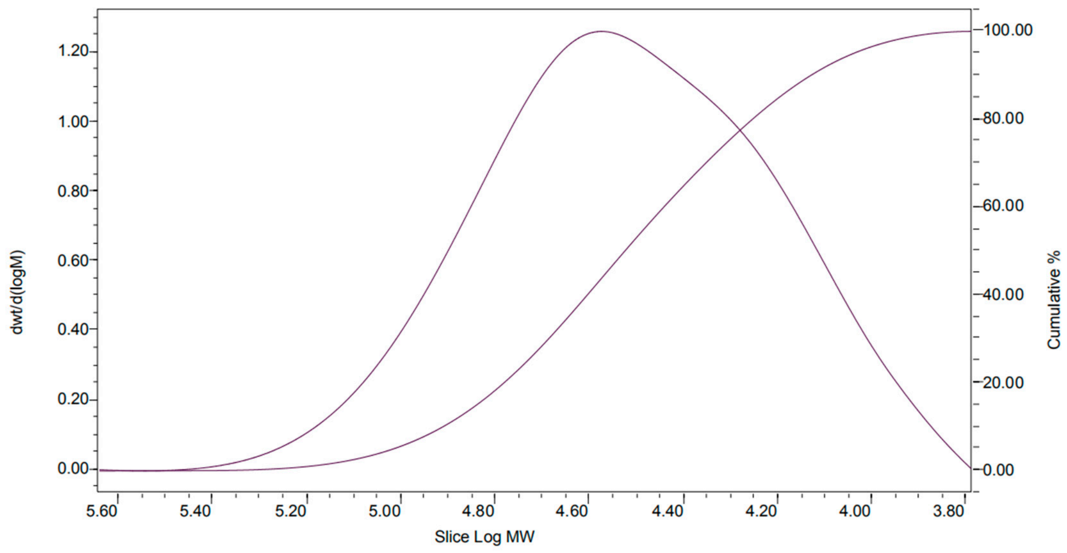

GPC Results

	Dist Name	Elution Volume (ml)	Retention Time (min)	Adjusted RT (min)	Mn	Mw	MP	Mz	Mz+1
1		23.392	23.392	23.392	26729	42178	37747	65244	93482

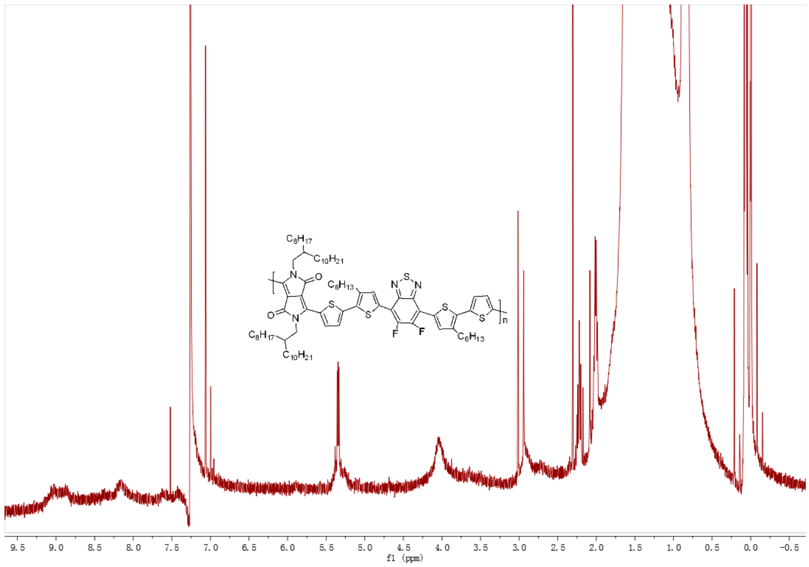

Figure S2 GPC traces and ^{1}HNMR of P2

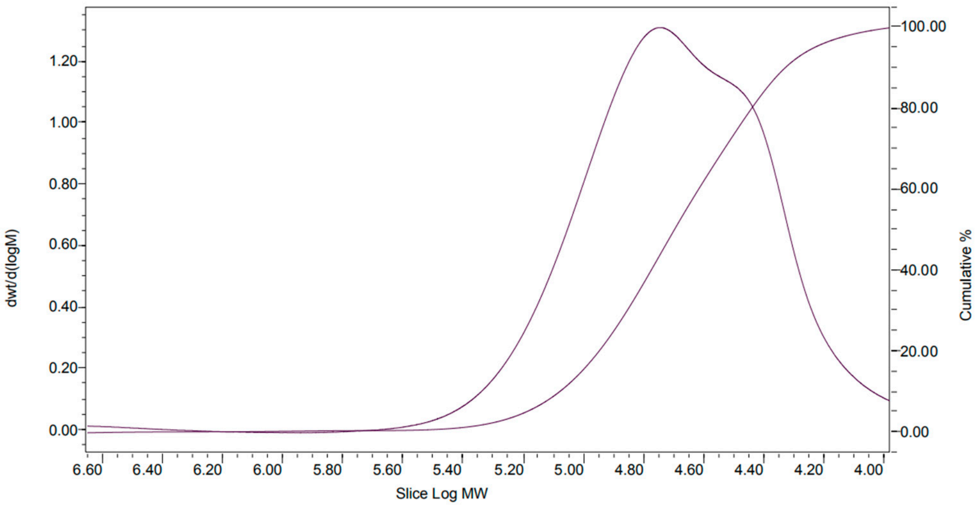

GPC Results

	Dist Name	Elution Volume (ml)	Retention Time (min)	Adjusted RT (min)	Mn	Mw	MP	Mz	Mz+1
1		15.898	15.898	15.898					
2		23.432	23.432	23.432	40523	62662	57013	97155	143904

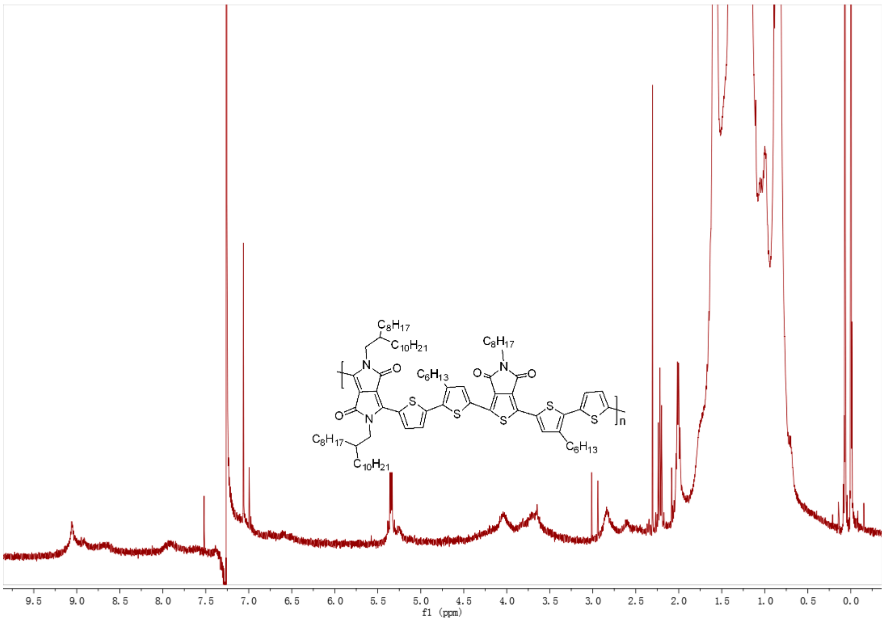

Figure S3 GPC traces and ^{1}HNMR of P3

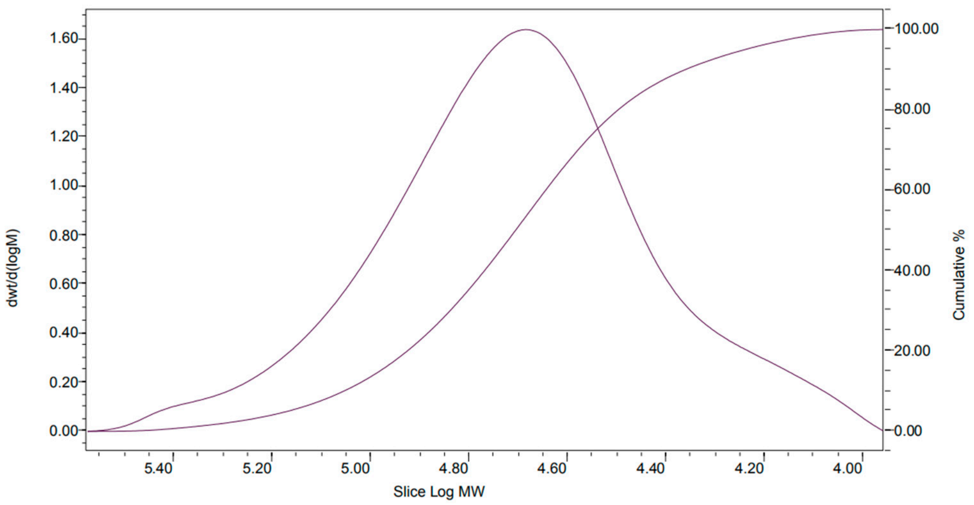

GPC Results

	Dist Name	Elution Volume (ml)	Retention Time (min)	Adjusted RT (min)	Mn	Mw	MP	Mz	Mz+1
1		22.917	22.917	22.917	42232	62171	48623	92234	132830

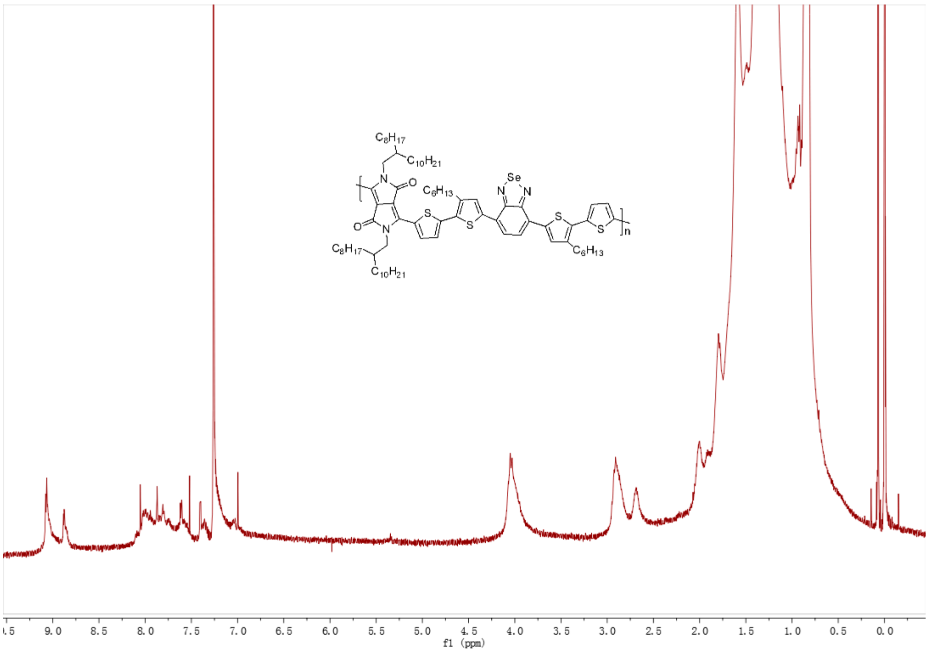

Figure S4 GPC traces and ^{1}HNMR of P4

1.3 AFM 3D images and corresponding values of blend films

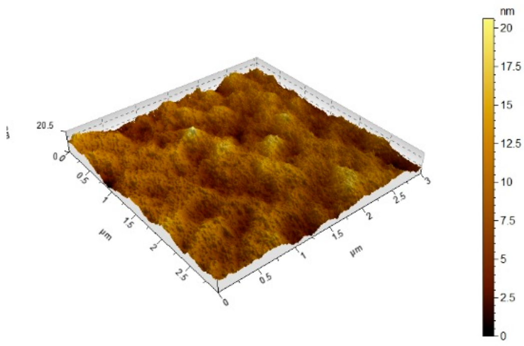

ISO 25178		
Height Parameters		
Sq	2.62	nm
Ssk	0.186	
Sku	3.05	
Sp	12.1	nm
Sv	8.52	nm
Sz	20.6	nm
Sa	2.09	nm

Figure S5 P1:$PC_{71}BM$ blend film

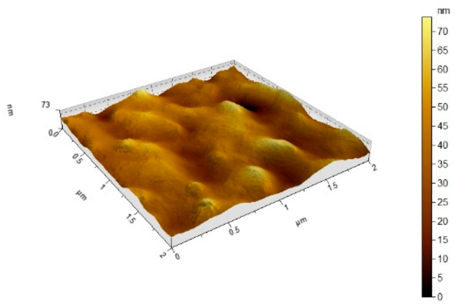

ISO 25178		
Height Parameters		
Sq	11.3	nm
Ssk	-0.0813	
Sku	2.97	
Sp	35.4	nm
Sv	38.3	nm
Sz	73.7	nm
Sa	9.02	nm

Figure S6 P2:$PC_{71}BM$ blend film

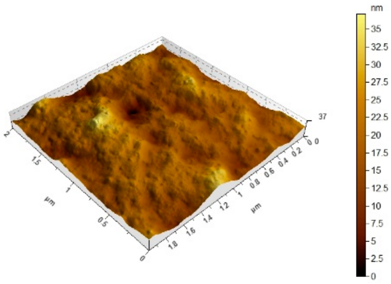

ISO 25178		
Height Parameters		
Sq	4.71	nm
Ssk	0.250	
Sku	3.56	
Sp	18.9	nm
Sv	18.3	nm
Sz	37.2	nm
Sa	3.68	nm

Figure S7 P3:$PC_{71}BM$ blend film

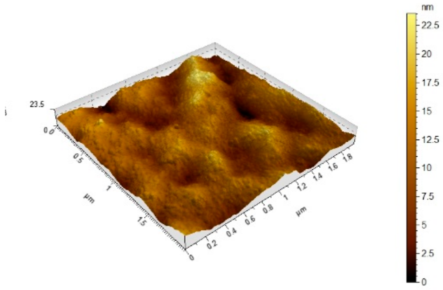

ISO 25178		
Height Parameters		
Sq	3.86	nm
Ssk	0.221	
Sku	2.87	
Sp	13.6	nm
Sv	9.96	nm
Sz	23.5	nm
Sa	3.09	nm

Figure S8 P4:$PC_{71}BM$ blend film
